# Influence of aldo–keto reductase 1C3 polymorphisms in early-onset female psoriasis patients

**DOI:** 10.1038/s41598-023-30464-8

**Published:** 2023-02-25

**Authors:** Yuka Nojiri, Motoki Nakamura, Tetsuya Magara, Aya Yamamoto, Kyoko Ikumi, Reiko Nakamura, Emi Nishida, Thomas Haarmann-Stemmann, Akimichi Morita

**Affiliations:** 1 Department of Geriatric and Environmental Dermatology, Nagoya City University Graduate School of Medical Sciences, Nagoya, Japan; 2 Department of Dermatology, Okazaki City Hospital, Okazaki, Japan; 3 IUF – Leibniz Research Institute for Environmental Medicine, Düsseldorf, Germany

**Keywords:** Genetics, Diseases, Pathogenesis, Risk factors

## Abstract

The principal pathology of psoriasis is impaired skin barrier function, epidermal thickening, and granular layer loss. Exposure to extrinsic factors such as tobacco smoke and air pollutants is associated with the development of psoriasis. Aryl hydrocarbon receptors (AHRs) are activated by extrinsic factors associated with the development of psoriasis and act as transcriptional regulators. Expression of aldo–keto reductase (AKR) 1C3 in the epidermal spinous layer regulates epidermal keratinocyte differentiation via the AHR signaling pathway. We investigated whether single nucleotide polymorphisms (SNPs) in AKR1C3 are associated with the pathogenesis of psoriasis. The proportions of rs12529 G/C, C/C variants, and rs12387 A/A, A/G variants were twofold higher in Japanese psoriasis patients (n = 231) compared with a Japanese healthy cohort. The SNPs were significantly more common than the majority variants in female patients with disease onset ≤ 22 years of age. Patients with rs12529 G > C and rs12387 A > G SNPs exhibited significantly lower AKR1C3 expression and higher expression of late differentiation markers. In conclusion, AKR1C3 downregulation caused by rs12529 G > C and rs12387 A > G SNPs in the epidermis induces abnormal early differentiation of keratinocytes and skin barrier dysfunction, which may contribute to the genetic pathogenesis of psoriasis in young females.

## Introduction

Psoriasis is an inflammatory disease in which T cells play important roles. T-helper (Th) -1, Th-17, and Th-22 interact with dendritic cells, macrophages, and keratinocytes, with the production of cytokines involved in the pathogenesis of psoriasis. Various therapeutic agents, including biologics, have been developed to inhibit such T cell functions with successful results^1^. The pathogenesis of psoriasis cannot be accounted for by immune response abnormalities alone, however, and an understanding of the genetic background is also essential^2^. The genetics of psoriasis have been examined in twin samples and patients with familial psoriasis^3,4^, and many genome-wide association analyses have been performed^5^. Currently, more than 80 loci associated with disease susceptibility have been identified, but the genetic background of psoriasis remains unclear^6^.

Exposure to extrinsic factors such as tobacco smoke and air pollutants is associated with the development of psoriasis. Smoking increases the risk of developing psoriasis as well as pustulosis^2,7^. Blood levels of cadmium, a typical air pollutant, were reported to be higher in patients with severe psoriasis^8^. The extrinsic factors act as ligands for the aryl hydrocarbon receptor (AHR) and activate the AHR signaling pathway. Activation of AHR signaling promotes epidermal terminal differentiation as well as filaggrin and loricrin expression, thereby restoring normal skin barrier function^9^. This skin barrier-restoring function of AHR has been applied for the treatment of psoriasis and atopic dermatitis^10^.

Aldo–keto reductase (AKR) 1C3 expression depends on AHR signaling^11^. AKR1C3 is a nicotinamide adenine dinucleotide phosphate (NADPH)-dependent oxidoreductase involved in prostaglandin (PG) D_2_ metabolism^12^. AKR1C3 is expressed in the epidermal spinous layer and regulates keratinocyte proliferation and differentiation^13^. Atopic dermatitis lesions exhibit upregulated AKR1C3, enhanced keratin 10, and reduced loricrin expression^13^. AKR1C3 expression in psoriasis lesions has not been reported, however, but is downregulated according to NCBI GEO profiles (GSE13355). To explore the association between reduced AKR1C3 expression and the development of psoriasis, we focused on single nucleotide polymorphisms (SNPs) of AKR1C3.

We evaluated 2 AKR1C3 SNPs, rs12529 and rs12387, in 231 psoriasis patients to determine the association of these SNPs with the clinical data and differences in keratinocyte immunostaining. We also isolated and cultured epidermal keratinocytes with each SNP and generated 3-dimensional (3D) skin cultures to analyze AKR1C3 expression and keratinocyte differentiation.

## Results

### AKR1C3 SNPs are more common in psoriasis patients than in healthy populations and are more highly associated with psoriasis vulgaris than with psoriatic arthritis

The patient cohort comprised 231 Japanese psoriasis patients (171 males, 60 females) treated with biologics at Nagoya City University Hospital. The diagnosis of psoriasis was based on clinical manifestations and histopathology. Disease severity was assessed before treatments based on the Psoriasis Area and Severity Index (PASI) score^14^. In this cohort, the mean PASI score was 24.6. Of the 231 patients, 130 had psoriasis vulgaris (PV), 67 had psoriatic arthritis (PsA), 22 had generalized pustular psoriasis (GPP), 5 had erythrodermic psoriasis (EP), and 7 had coexisting PsA and GPP. The body mass index (BMI) of this cohort ranged from 15.6 to 44.1 (mean 25.6). The clinical characteristics of the cohort are summarized in Table 1.

**Table 1:** Patient characteristics and psoriasis types.

Characteristics		
Cases		231
Race		Japanese
Age at onset (range)		33.3 (0–79)
sex	Male	171
	Female	60
BMI at first visit (range)		25.6 (15.6–44.1)
PASI score before treatment (range)		24.6 (0.6–72)
Tobacco smoking	Ever smoker	99
	Never smoker	130
	unknown	2
Psoriasis type	PV	130
	PsA	67
	GPP	22
	EP	5
	PsA + GPP	7

*BMI* Body mass index, *PV* Psoriasis vulgaris, *PsA* Psoriatic arthritis, *GPP* Generalized pustular psoriasis, *EP* Erythrodermic psoriasis, *PASI* Psoriasis area and severity index.

A TaqMan genotyping assay was performed using DNA isolated from whole blood. Rs12529 and rs12387, 2 common AKR1C3 SNPs, were examined. The frequency of both SNPs is as low as 6% to 11% in healthy Japanese. The rs12529 SNP frequency in Japanese differs significantly from the global trend, which has an rs12529 G > C frequency of 42%. The TaqMan SNP genotyping assay revealed that the rs12529 genotype distribution in our cohort was G/G:174 (75.3%), G/C:52(22.5%), and C/C:5 (2.2%). Thus, the probability of a G > C mutation in rs12529 was 13.4%. Surprisingly, the A > G mutation in rs12387 and G > C mutation in rs12529 were always observed simultaneously. The proportions of rs12529 G > C SNPs and rs12387A > G SNPs were significantly higher in the patient cohort than in the 2 cohorts of healthy Japanese individuals in the NCBI database (rs12529, *p *= 0.0080 [*vs* ss69068306], *p *= 0.0029 [*vs* ss71643788], chi-square test, Table 2, and rs12387, *p *= 0.0093 [*vs* ss2827707], *p *= 0.0026 [*vs* ss66361131], chi-square test, Table 3). Compared with a larger cohort of Japanese called "8.3 K JPN”, which may also include a variety of diseases including psoriasis, the rs12529 G > C SNP tended to be more prevalent in psoriasis patients (*p *= 0.0535, chi-square test, Table 2)^15^. In rs12387, A > G SNPs were significantly more common in our cohort than in “8.3 K JPN” (*p *= 0.0363, chi-square test, Table 3).

**Table 2:** Genotyping results for rs12529 and comparison with other cohorts.

rs12529 Study	Population	Sample number	Major Allele G	Minor Allele C	Chi-square test *P* value
Our study	Japanese	231	400 (86.6%)	62 (13.4%)	
ss69068306	Japanese	90	168 (93.3%)	12 (6.7%)	0.0080
ss71643788	Japanese	88	166 (94.3%)	10 (5.7%)	0.0029
8.3KJPN	Japanese	16,760	29,816 (88.9%)	3704 (11.1%)	0.0535
1000Genomes	Global	5008	5797 (58.0%)	4203 (42.0%)

**Table 3:** Genotyping results for rs12387 and comparison with other cohorts.

rs12387 study	Population	Sample number	Major Allele A	Minor Allele G	Chi-square test *P* value
Our study	Japanese	231	400 (86.6%)	62 (13.4%)	
ss2827707	Japanese	172	316 (91.9%)	28 (8.1%)	0.0093
ss66361131	Japanese	90	168 (94.4%)	10 (5.6%)	0.0026
8.3KJPN	Japanese	16,760	29,898 (89.2%)	3622 (10.8%)	0.0363
1000Genomes	Global	5008	8482 (84.8%)	1518 (15.2%)	

**Figure 1:**
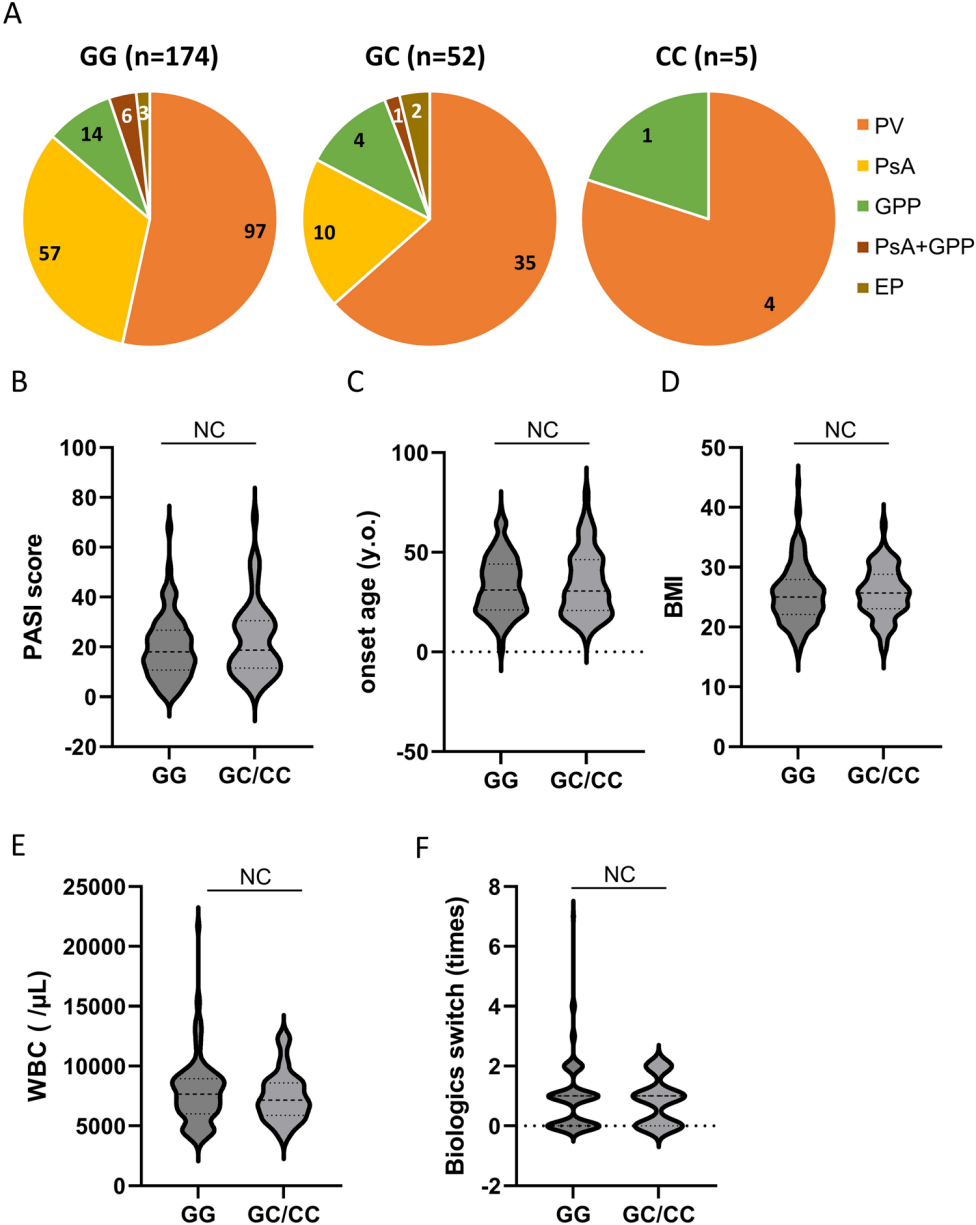
Patient characteristics and AKR1C3 SNPs. (**A**) Breakdown of psoriasis type for each single nucleotide polymorphism (SNP). Patients with the rs12529 G > C and rs12387 A > G SNPs had more psoriasis vulgaris (PV) and less psoriatic arthritis (PsA; *p *= 0.0386, Fisher’s exact test). No correlation was detected between AKR1C3 SNPs and (**B**) Psoriasis Area and Severity Index (PASI) score, (**C**) disease onset age, (**D**) body mass index (BMI), (**E**) white blood cell (WBC) count at the first visit, and (**F**) the number of times biologics were switched. *EP* Erythrodermic psoriasis, *GPP* Generalized pustular psoriasis, *EP* Erythrodermic psoriasis.

The group with the rs12529 G > C SNP, which also had the rs12387 A > G SNP, had more PV and less PsA (*p *= 0.0386, Fisher’s exact test, Fig. 1A). No correlation was detected between the AKR1C3 variant and smoking (data not shown), PASI score (Fig. 1B), onset age (Fig. 1C), BMI (Fig. 1D), white blood cell (WBC) count at first visit (Fig. 1E), or the number of times biologics were switched (Fig. 1F).

### AKR1C3 SNPs are related to early-onset psoriasis in female patients

**Figure 2:**
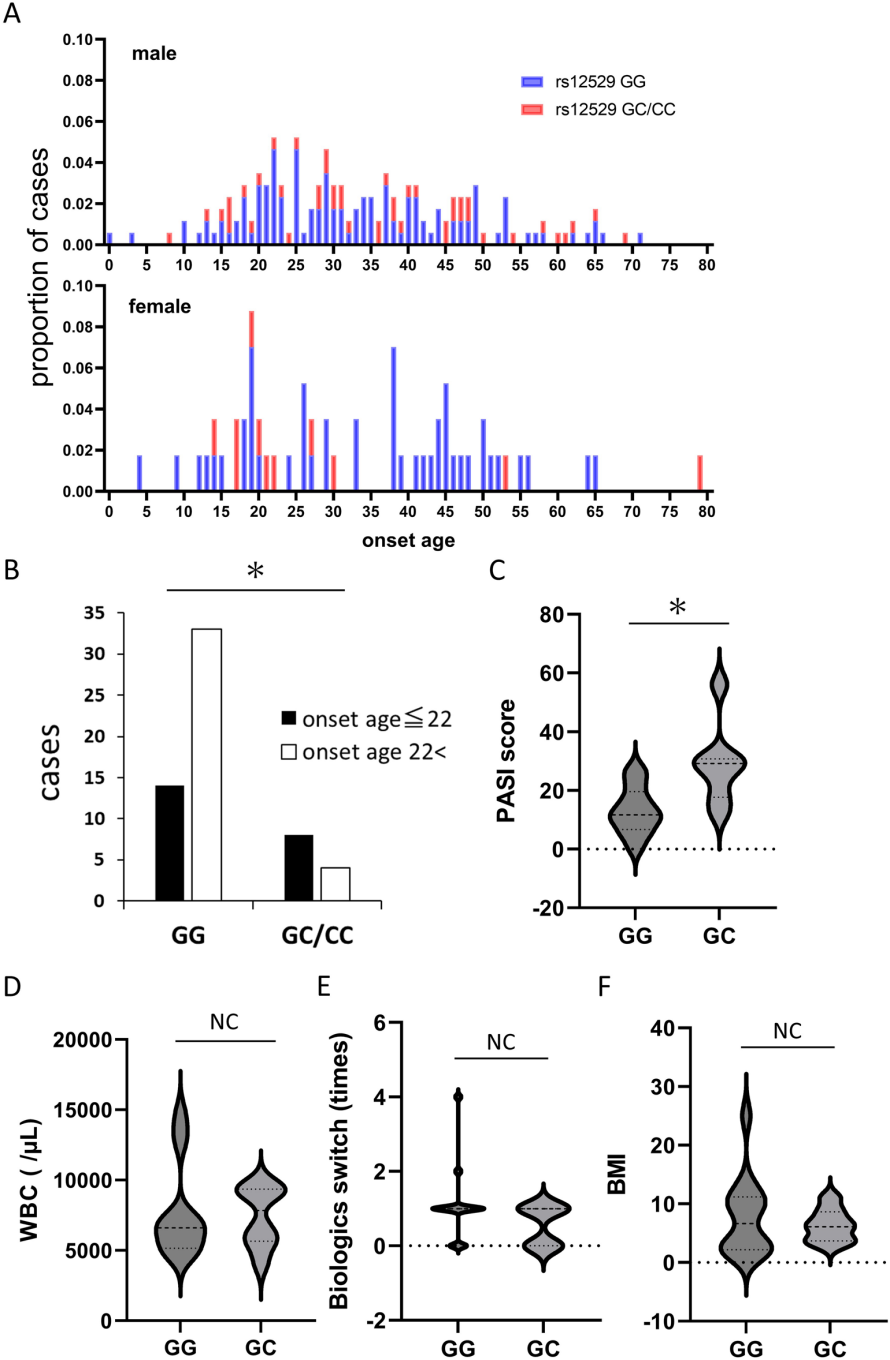
SNPs in young-onset female patients. (**A**) Distribution of age of onset by sex. The onset age in female patients showed bimodal peaks, with the first peak around 20 years of age and the second peak between 40 and 50 years of age. Female patients with rs12529 G > C and rs12387 A > G SNPs were more common in the first peak (< 22 years of age). (**B**) Significantly more female patients with an onset age ≤ 22 had the rs12529 G > C and rs12387 A > G variants compared with the rs12529 G/G and rs12387A/A variants (*p *= 0.0213, Fisher’s exact test). (**C**) Among young female patients whose onset age ≤ 22, patients with rs12529 G > C and rs12387 A > G had a significantly higher PASI score than those with rs12529 G/G and rs12387 A/A (*p *= 0.0063, Student’s t-test). No correlation was detected between clinical features, including (**D**) white blood cell (WBC) count, (**E**) the number of times biologics were switched, and (**F**) body mass index (BMI).

The mean onset age in the cohort was 33.3 years (Table 1), and did not differ significantly between males (33.7 years) and females (32.7 years); the distribution, however, was very different (Fig. 2A). As in previous reports^16,17^, the onset age in female patients had bimodal peaks, with the first peak around 20 years of age and the second peak between 40 and 50 years of age. Female patients with rs12529 G > C and rs12387 A > G SNPs were more common in the first peak (> 22 years of age). The number of female patients with an onset age ≤ 22 having the rs12529 G/C, C/C, and rs12387A/G, G/G variants was significantly higher than that of female patients having the rs12529 G/G and rs12387A/A SNPs (*p *= 0.0213, Fisher’s exact test, Fig. 2B). Young female patients with an onset age of ≤ 22 having rs12529 G > C and rs12387 A > G SNPs had significantly higher PASI scores than those with rs12529 G/G and rs12387 A/A SNPs (*p *= 0.0063, Student’s test, Fig. 2C). No correlation was detected between clinical features, including WBC count (Fig. 2D), number of times that biologics were switched (Fig. 2E), BMI (Fig. 2F), and AKR1C3 variants, in young female patients or the whole cohort.

### AKR1C3 expression in the psoriasis lesional epidermis is decreased in patients with AKR1C3 SNPs

**Figure 3:**
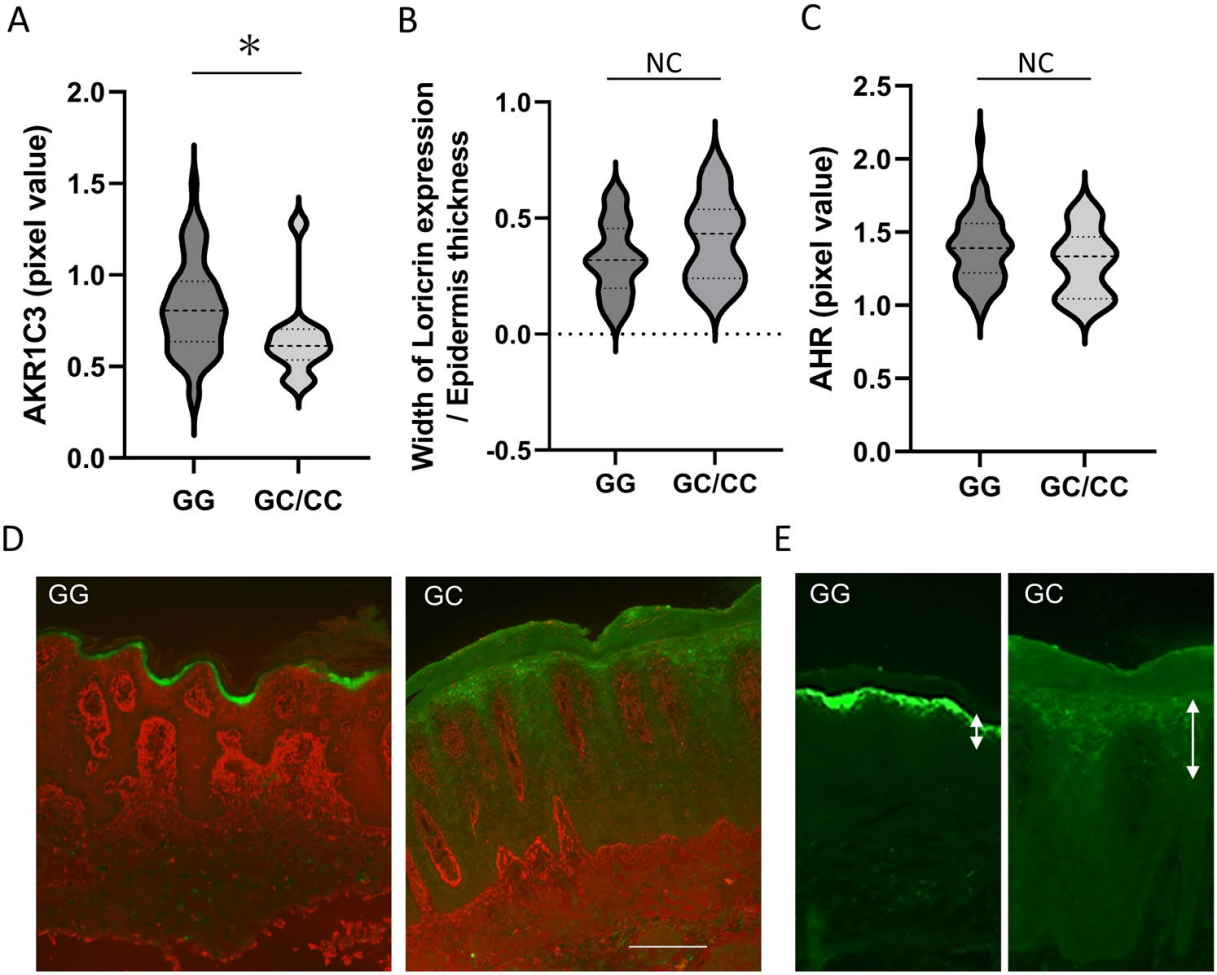
Results of immunohistochemistry. Immunostaining for AKR1C3 and loricrin was performed on formalin-fixed paraffin-embedded tissues of psoriasis lesions from 62 patients. (**A**) AKR1C3 expression in the epidermis was significantly lower in psoriasis lesional skin samples from patients with rs12529 G > C and rs12387 A > G than in patients with rs12529 G/G and rs12387 A/A (*p *= 0.0434, Student’s t-test). (**B**) Wide loricrin expression due to abnormal early expression was observed in the epidermis from patients with rs12529 G > C and rs12387 A > G SNPs. (**C**) AHR expression did not differ depending on the variant. (**D**) Representative immunostaining images of AKR1C3 (red) and loricrin (green) staining. (**E**) Ectopic expression of loricrin in the spinous layer in patients with the rs12529 G > C SNP.

Immunostaining of AKR1C3 and loricrin was performed on formalin-fixed paraffin-embedded tissues of psoriasis lesions obtained from 62 of 231 patients (45 males, 17 females; 28 patients with PV, 19 with PsA, 11 with GPP, 1 with EP, 3 with complications). AKR1C3 expression (red) in the epidermis from psoriasis lesional skin samples was significantly lower in patients with rs12529 G > C and rs12387 A > G SNPs than in patients with rs12529 G/G and rs12387 A/A SNPs (*p *= 0.0434, Student’s t-test, Fig. 3A, D). As for loricrin expression (green) in the epidermis, abnormally early and weak, but the broad expression was observed in the epidermis from patients with rs12529 G > C and rs12387 A > G SNPs (Fig. 3B, E). Expression of AHR, which induces AKR1C3^11^, also did not differ significantly between patients with different SNPs (Fig. 3C). Representative immunostaining images of AKR1C3 and loricrin staining are shown in Fig. 3D. In the major allele (rs12529 GG), AKR1C3 expression (red) in the epidermis was found in the suprabasal layers, not in the basal layer, as in a previous report^13^. The ectopic expression of loricrin (green) in the spinous layer in patients with the rs12529 G > C SNP is shown in Fig. 3E.

### AKR1C3 is downregulated and loricrin is upregulated in cells isolated from patients with AKR1C3 SNPs

**Figure 4:**
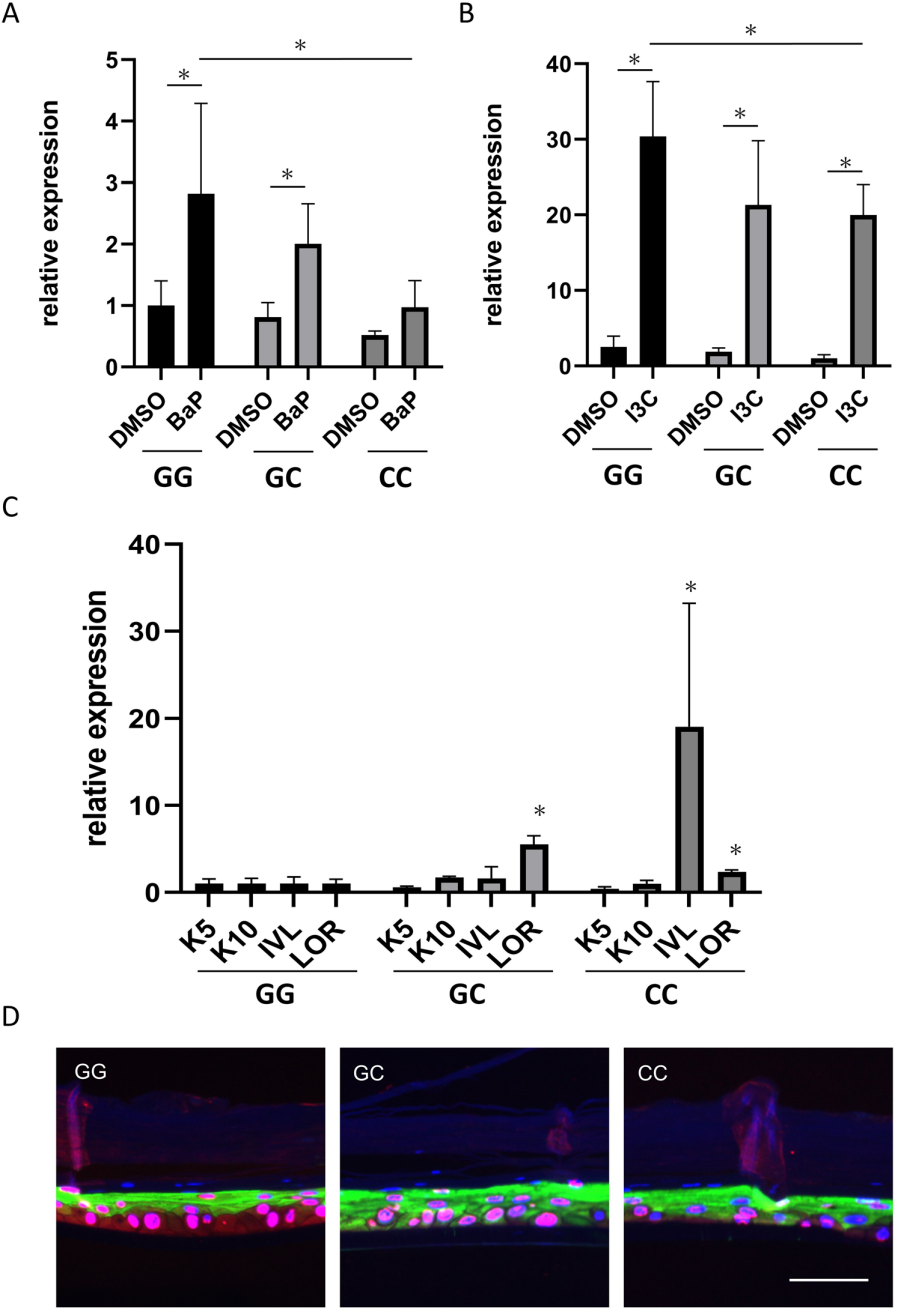
Cell culture isolated from each AKR1C3 SNPs. AKR1C3 expression in cells isolated from patients with each SNP was quantified by real-time polymerase chain reaction (**A**) after treatment with BaP (2.5 µM) and (**B**) after treatment with I3C (200 µM). AKR1C3 expression was significantly decreased in cells with the rs12529 G > C or rs12387 A > G mutations. (**C**) Expression of keratinocyte differentiation-related genes in real-time PCR of cells derived from each SNP. K5, keratin 5; K10, keratin 10; IVL, involucrin; LOR, loricrin. Cells isolated from patients with rs12529 G > C and rs12387 A > G mutations had increased involucrin and loricrin expression. (**D**) Immunostaining of AKR1C3 (red) and loricrin (green) in the 3D skin culture constructed with keratinocytes isolated from patients with each AKR1C3 SNP. Abnormal early expression of loricrin from the basal layer was observed in the skin cultures obtained from patients with rs12529 G > C and rs12387 A > G.

We isolated epidermal keratinocytes from patients with AKR1C3 rs12529 G/G, G/C, C/C, and rs12387 A/A, A/G, G/G, and analyzed them with 2D skin culture. After 6 days of treatment with 2.5 µM benzo[a]pyrene (BaP), a typical air pollutant and AHR agonist, and 200 µM indole-3-carbinol (I3C, an AHR agonist that specifically induces AKR1C3), AKR1C3 expression was significantly higher in keratinocytes with rs12529 G/G than in keratinocytes with rs12529 C/C (with BaP, *p *= 0.0009, Dunnett’s test, Fig. 4A, with I3C, *p *= 0.0353 Dunnett’s test, Fig. 4B) by real-time polymerase chain reaction (PCR). Expression of genes related to keratinocyte differentiation after 6-day treatment with 200 μM I3C was analyzed, and expression of genes associated with late differentiation, including involucrin and loricrin, was significantly upregulated in keratinocytes obtained from patients with rs12529 G > C and rs12387 A > G SNPs, despite using 2D cultures with no air–liquid interface (*p *= 0.0495, Wilcoxon test, Fig. 4C). Interestingly, rs12529 G/C-derived keratinocytes exhibited high loricrin expression, while rs12529 C/C-derived keratinocytes exhibited reduced loricrin expression and the highest involucrin expression.

### Abnormal early expression of loricrin observed in 3D skin culture of keratinocytes from patients with AKR1C3 SNPs

The 3D skin culture was constructed with keratinocytes isolated from patients with each of the AKR1C3 SNPs. The incubation period was 2 weeks; for the first 7 days, keratinocytes were incubated in the medium supplied with the EPI-KIT (J-TEC, Gamagori, Japan), and for the second 7 days, they were incubated in the medium supplemented with 300 μM I3C. Keratinocytes were fixed on day 14 and immunohistochemical staining was performed. Abnormally early expression of loricrin (green staining) from the basal layer was observed in the skin cultures obtained from patients with rs12529 G/C and C/C SNPs (Fig. 4D).

### Expression landscape of genes associated with keratinocyte differentiation in psoriasis

**Figure 5:**
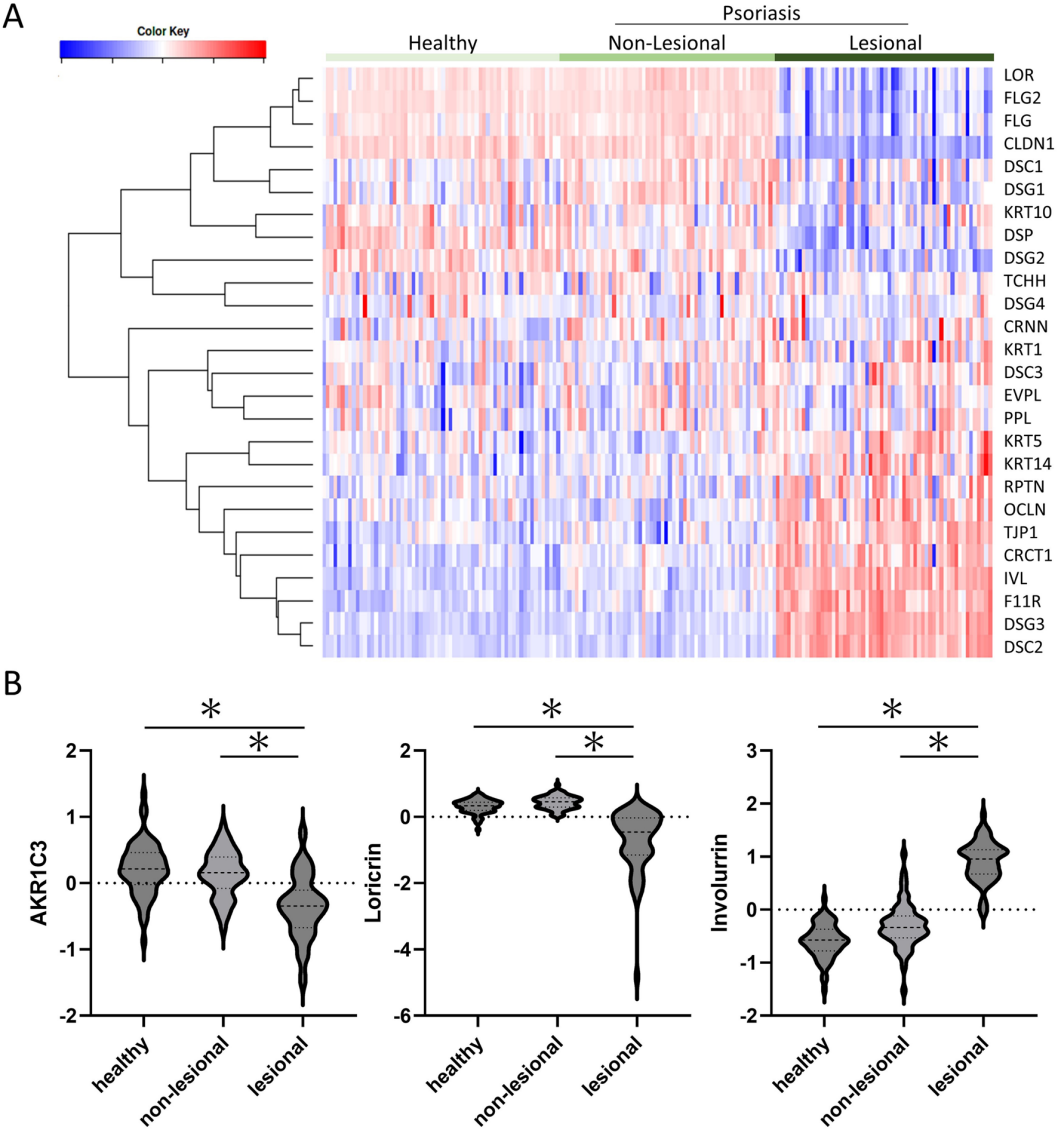
Analysis using the profiled data set. Analysis was performed using the profiled data set containing 180 samples accessible at the GEO database (GSE13355). (**A**) Clustering heat map of genes related to keratinocyte differentiation and skin barrier construction. (**B**) Comparison of AKR1C3 expression with loricrin and involucrin expression in the same data set. *LOR* Loricrin, *FLG* Filaggrin, *CLDN1* Claudin-1, *DSC* Desmocollin, *DSG* Desmoglein, *KRT* Keratin, *DSP* Desmoplakin, *TCHH* Trichohyalin, *CRNN* Cornulin, *EVPL* Envoplakin, *PPL* Periplakin, *RPTN* Repetin, *OCLN* Occludin, *TJP1* Tight junction protein 1, *CRCT1* Cysteine-rich C-terminal 1, *IVL* Involucrin, *F11R* F11 receptor.

Analysis was performed using the profiled data set accessible at the GEO database (GSE13355)^18^. Clustering analysis revealed genes that were downregulated and upregulated in psoriasis lesions (Fig. 5A). Loricrin (LOR) was the most significantly downregulated gene in the psoriasis lesions, and filaggrin (FLG, FLG2) was also strongly downregulated. Involucrin (IVL), on the other hand, was upregulated. Keratin 5 (KRT5) was highly expressed while keratin 10 (KRT10) was suppressed. Although tight junction protein 1 (TJP1) and occludin (OCLN) were strongly expressed in lesional skin, the absence of claudin (CLDN1) expression indicates that tight junctions were not well formed. In the psoriasis lesional skin, high expression of desmocollin 2 (DSC2) and desmoglein 3 (DSG3) was also observed, in contrast to the low expression of desmocollin 1 (DSC1) and desmoglein 1 (DSG1). The psoriasis lesional skin was characterized by increased gene expression in the lower to the middle epidermis and decreased gene expression related to terminal differentiation in the upper layer of the epidermis and granular layer and cornification. The expression of AKR1C3 was also included in this data set. In the psoriasis lesional skin, the expression of AKR1C3 was significantly downregulated compared with that in healthy normal skin and non-lesional skin (Fig. 5B). Although the number of AKR1C3 SNPs in this cohort is unknown, keratinocyte differentiation was clearly abnormal in the psoriasis lesional skin. This database differs from our Japanese cohort in that it comprises a US cohort. As described above, however, the rs12529 G > C SNP is much more common in the US, even in healthy individuals.

## Discussion

**Figure 6:**
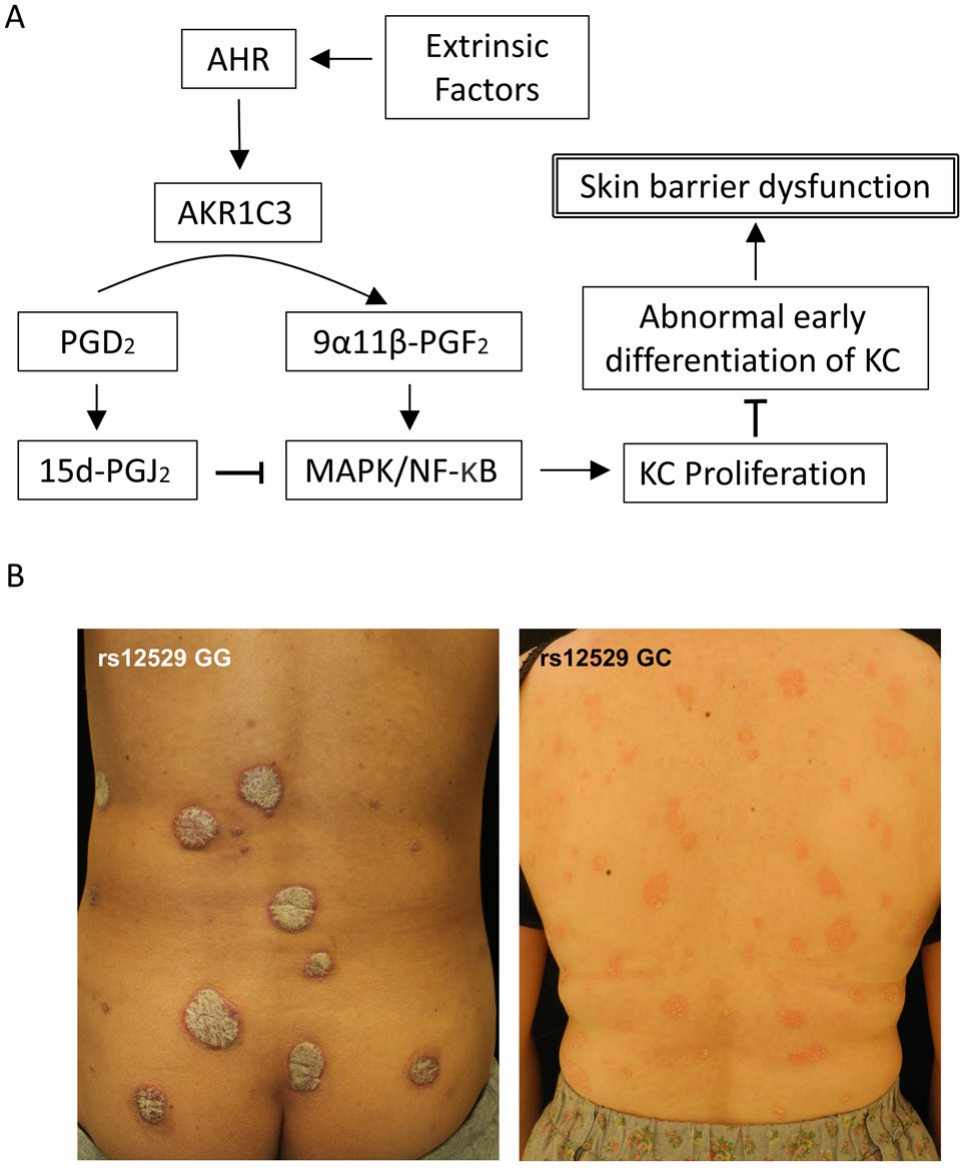
Association of AKR1C3 with keratinocyte proliferation and differentiation. (**A**) Schema showing the association of AKR1C3 with keratinocyte proliferation and differentiation. (**B**) Typical clinical presentation of patients with each SNP.

We found that rs12529 G > C and rs12387 A > G SNPs in AKR1C3, an enzyme involved in prostaglandin metabolism and keratinocyte differentiation, are more common in female psoriasis patients with early disease onset and may be associated with severe disease. These SNPs downregulate AKR1C3 expression in the psoriasis lesional epidermis and impair keratinocyte AKR1C3-mediated regulation of keratinization. This may increase disease susceptibility to psoriasis. Early-onset female psoriasis patients are unrelated to those with adult-onset diseases, such as obesity or hyperlipidemia. This finding provides a new concept of disease susceptibility due to epidermal genetic factors in psoriasis that could point to preventive methods and new therapeutic agents. AKR1C3 regulates cell proliferation and differentiation; AKR1C3 converts prostaglandin (PG) D2 to 9α11β-PGF_2_^12^, which acts on PGF receptors and activates the mitogen-activated protein kinase (MAPK) cascade signaling pathway to promote cell proliferation^19^. Downregulation of AKR1C3 expression in the epidermis by SNPs decelerates the metabolism of PGD_2_ to 9α11β-PGF_2_ and inhibits the MAPK cascade and nonenzymatic conversion of unmetabolized PGD_2_ to 15d-PGJ2, which has antiproliferative effects via MAPK inhibition, indirectly promoting abnormal early differentiation of keratinocytes and ectopic expression of skin barrier proteins. This epidermal change leads to skin barrier dysfunction (Fig. 6A). The pathologic characteristics of psoriasis are disruption of the skin barrier function due to aberrant proliferation and impaired differentiation of keratinocytes^20^. Abnormal early differentiation of epidermal keratinocytes associated with reduced expression of AKR1C3 may be one aspect of the pathogenesis of psoriasis.

As noted above, the onset age of psoriasis in women has a bimodal distribution^16,17^. This trend is similar not only in Asia but also worldwide. Psoriasis is a more common disease in males, but in young (pediatric) onset cases, females are slightly more likely to develop the disease^21–23^. Young-onset psoriasis has a smaller and thinner rash than adult-onset psoriasis^21,22,24^. This may be due to downregulated AKR1C3 in the epidermis, which suppresses cell proliferation. Clinical photographs of representative cases with and without AKR1C3 SNPs are shown in Fig. 6B. In these cases, patients with rs12529 GG SNP have thick, hard, and fixed skin lesions, whereas patients with rs12529 GC SNP have thin, soft, and easily moved skin lesions. Whereas rs12529 GG is less likely to change symptoms, rs12529 GC is prone to rapid aggravation such as erythrodermatization. This was supported by the higher PASI score of patients with the rs12529 G > C SNP in young-onset female patients. Since AKR1C3 is AHR-dependent, SNPs in AKR1C3 may define response to treatment with AHR agonists such as tapinarof^10^.

In the psoriasis patient cohort in this study, 2 SNPs, rs12529 and rs12387, were always found in the same patient. Rs12529 is one of the most common SNPs in AKR1C3. As described above, this SNP is rare in Asia (~ 10%), but it has a high global incidence (> 40%). Especially in Europe and Africa, the major allele and minor allele are reversed; more than 50% of the population has a G > C mutation^25^. The very large frequency differences by race also suggest that genetic factors are most likely responsible for the acquisition of this SNP. We also suspected the involvement of environmental factors, but found no differences in exposure to environmental factors, including smoking rates, in our cohort. Rs12529 is reported to be associated with prostate and bladder cancer^26,27^. AKR1C3 catalyzes the synthesis of androgens^28^ and thus the function of AKR1C3 may differ depending on the SNP. Particularly in prostate cancer, AKR1C3 is strongly expressed in the tumor and is expected to be a biomarker and therapeutic target^29,30^. Rs12529 is also reported to be associated with smoking habit^26^. The effect of SNPs on AKR1C3 function remains inconclusive. Shiota et al. have shown that the H5Q mutation caused by rs12529 has only a minor effect on AKR1C3 activity and protein stability^31^, and similar results were obtained by Detlefsen et al. ^32^. However, our recent report showed that the G > C mutation in rs12529 (major allele in Europe) resulted in less protein stability compared to the major allele in Asia^33^. On the other hand, the ratio of major allele (A) to minor allele (G) in rs12387 is almost the same worldwide, and A > G mutations are found in approximately 15% of cases. In Japan, the frequency is slightly lower (10.8%)^15^. Rs12387 is also associated with the development of breast cancer in women who underwent combined estrogen-progesterone therapy^34^ or were exposed to polycyclic aromatic hydrocarbons^35^. The involvement of AKR1C3 hormone metabolism and the AHR signaling pathway is suspected, but the mechanism remains unclear. Based on the present findings, it is unclear which SNP is at work in association with psoriasis disease susceptibility, maybe both. As noted above, the probabilities of the 2 SNPs vary widely worldwide but are similar in East Asia. It may be a feature only in East Asia that psoriasis patients have the 2 SNPs. Further analyses of SNPs in worldwide cohorts as well as functional analyses of each SNP are needed.

## Methods

### Patient cohort

The patient cohort comprised 231 Japanese psoriasis patients treated at Nagoya City University Hospital between 2015 and 2019. Psoriasis was diagnosed by clinical findings and pathology, and all patients who consented to blood sampling were analyzed. PASI scoring is performed by several dermatologists with more than 7 years of clinical experience and is standardized within the facility at conferences. All included patients had been treated with biologics and had had more severe psoriasis than the general population. All experiments were initiated after receiving approval from the institutional review board (Nagoya City University Clinical Trial Management Center, No. 70–00-0100). All research was performed under the declaration of Helsinki, and informed consent was obtained from all participants or their legal guardians.

### SNP genotyping assay

Genomic DNA was extracted from 100 µl whole blood using the DNeasy Blood & Tissue Kit (Qiagen, Venlo, the Netherlands) according to the manufacturer’s protocol, and stored at − 20 °C. The 2 SNPs in AKR1C3, rs12529 G > C and rs12387 A > G, were analyzed by a TaqMan SNP genotyping assay (Thermo Fisher Scientific, Waltham, MA). Sequences of TaqMan MGB probes/extension primers were summarized in supplementary table 1. The total PCR reaction volume contained 1.0 ng/μl genomic DNA, 5.0 μl TaqPath ProAm Master Mix (Thermo Fisher Scientific), and 0.5 μl of 20 × SNP assay mix, and was adjusted to a final volume of 10 μl using nuclease-free water. PCR was performed by Applied Biosystems 7500HT Fast Real-Time PCR System (Thermo Fisher Scientific), under the following conditions: pre-reading at 25 °C for 30 s, initial enzyme activation at 95 °C for 5 min, followed by 40 cycles of amplification; denaturation at 95 °C for 5 s, annealing/extension for 30 s at 60 °C, finally post reading at 25 °C for 30 s. Fluorescence data collection was performed at the annealing/extension step for FAM and VIC dye.

### Immunohistochemical staining

Tissues were obtained by biopsy from patients and fixed in 10% formalin before embedding in paraffin. Sections were deparaffinized in xylene for 10 min, followed by graded rehydration in EtOH (99.5%, 95%, 85%, 70%, and 50%) for 5 min each. For AKR1C3 and loricrin, sections were incubated in antigen retrieval buffer (10 mM Tris, 1 mM EDTA, 0.05% Tween-20, pH 6.0) at 95 °C for 10 min in a water bath. For AHR, sections were incubated in antigen retrieval buffer (10 mM Tris, 1 mM EDTA, pH 9.0) at 95 °C for 10 min in a pressure cooker. Sections were incubated in the following primary antibodies, 1:46 anti-AKR1C3 antibody (Sigma-Aldrich, Darmstadt, Germany), 1:500 anti-loricrin antibody (Abcam, Cambridge, UK), or 1:50 anti-AHR antibody (Invitrogen, Waltham, MA) overnight at 4 °C. Sections were then incubated in 1:500 Alexa Fluorescein 594-conjugated goat anti-mouse IgG antibody (Invitrogen), 1:500 Alexa Fluorescein 488-conjugated secondary goat anti-rabbit IgG antibody (Invitrogen) for 30 min at 37 °C. The images were obtained using a fluorescence microscope BZ-X810 (Keyence, Osaka, Japan). The fluorescence intensities of AKR1C3 and AHR were calculated using ImageJ software (NIH, Bethesda, MD) from 10 randomly selected fields.

### Cell culture

The primary culture of human epidermal keratinocytes was kindly supported by J-TEC (Gamagori, Japan). Keratinocytes were isolated from 3 patients with each of the 3 types of SNPs in AKR1C3; rs12529 GG and rs12387 AA, rs12529 GC and rs12387 AG, and rs12529 CC and rs12387 GG. Skin tissue obtained from the patients was isolated as individual cells and proliferated according to a protocol using 3T3 cells as feeders^36^. The completed cell lines were incubated in HuMedia-KG2 medium supplemented with 0.1% insulin, 0.1% human epidermal growth factor, 0.1% hydrocortisone, 0.4% bovine pituitary extract, and 0.1% gentamicin/amphotericin B (Kurabo, Osaka, Japan).

### RNA isolation and real-time PCR

As an AHR agonist, I3C (AdipoGen, Liestal, Switzerland) and BaP (MilliporeSigma, St. Louis, MO) were used as pretreatments. Total RNA was extracted from the cultured keratinocytes with an RNeasy Mini Kit (Qiagen, Hilden, Germany). Total RNA was reverse transcribed into complementary DNA using a T100 thermal cycler (Bio-Rad, Hercules, CA). Real-time PCR was performed using CFX Connect real-time System (Bio-Rad). The gene-specific forward and reverse primer sequences used were summarized in supplementary table 1.

### 3D skin cultures

The 3D skin culture was constructed using an EPI-KIT (J-TEC) by replacing the attached normal human epidermal keratinocytes with keratinocytes isolated from patients with each of the AKR1C3 SNPs. The incubation period was 2 weeks, with the first 7 days incubated in the medium supplied by J-TEC and the second 7 days in the medium supplemented with 300 μM of I3C. After 14 days of culture, the skin culture tissue was fixed in 10% formalin and embedded in paraffin.

### In silico* analysis*

In silico analysis was performed using the profiled data set containing 180 samples accessible at the GEO database (GSE13355). A clustered heatmap of all samples was generated using the online tool iDEP.91 (http://bioinformatics.sdstate.edu/idep/)^37^.

### Statistical analysis

Statistical analyses were performed using GraphPad Prism version 9.5.0 for Windows (GraphPad Software, San Diego, CA, www.graphpad.com) and Pharmaco Basic (Three S, Tokyo, Japan). Probability values less than 0.05 were considered statistically significant.

## Supplementary Information

Supplementary Information.

## Data Availability

The datasets used and analyzed during the current study are available from the corresponding author upon reasonable request.
