# Supplementary material for: Influence of aldo–keto reductase 1C3 polymorphisms in early-onset female psoriasis patients
Source: Sci Rep. 2023 Feb 25;13:3280. doi: 10.1038/s41598-023-30464-8 (PMC9968313; doi:10.1038/s41598-023-30464-8)
Supplement: Supplementary file 1 — Supplementary Information. [file 41598_2023_30464_MOESM1_ESM.docx]

Supplementary Table 1

Primer sequences

The following primer sequences were used in this study.

| TaqMan probe | | |
| --- | --- | --- |
| rs12529 | VIC | 5’-AGTGACAGGGAATGGATTCCAAACA-3’ |
|  | FAM | 5’-CAGTGTGTAAAGCTAAATGATGGCC-3’ |
| rs12387 | VIC | 5’-GACCAGCCTTGGAAAACTCACTGAA-3’ |
|  | FAM | 5’-AAAGCTCAATTGGACTATGTTGACC-3’ |
| qRT-PCR primer | | |
| AKR1C3 | forward | 5’-GTTGCCTATAGTGCTCTGGGATCT-3’ |
|  | reverse | 5’-GGACTGGGTCCTCCAAGAGG-3’ |
| Keratin 5 | forward | 5’-ATCGCCACTTACCGCAAGCTGCTGGAGGG-3’ |
|  | reverse | 5’-AAACACTGCTTGTGACAACAGAG-3’ |
| Keratin 10 | forward | 5’-ATGCCAACATCCTGCTTCAGAT-3’ |
|  | reverse | 5’-GCAGTGCTACCTCATTCTCATACTTC-3’ |
| Involucrin | forward | 5’-TCCTCCTCCAGTCAATACCC-3’ |
|  | reverse | 5’-GCTGATCCCTTTGTGTT-3’ |
| Loricrin | forward | 5’-TCATGATGCTACCCGAGGTTTG-3’ |
|  | reverse | 5’-CAGAACTAGATGCAGCCGGAGA-3’ |
